# Resident Macrophages and Their Potential in Cardiac Tissue Engineering

**DOI:** 10.1089/ten.teb.2021.0036

**Published:** 2022-06-10

**Authors:** Meenakshi Suku, Lesley Forrester, Manus Biggs, Michael G. Monaghan

**Affiliations:** ^1^ Department of Mechanical, Manufacturing and Biomedical Engineering, Trinity College Dublin, Dublin, Ireland.; ^2^ Trinity Centre for Biomedical Engineering, Trinity Biomedical Science Institute, Trinity College Dublin, Dublin, Ireland.; ^3^ CURAM SFI Research Centre for Medical Devices, National University of Ireland, Galway, Ireland.; ^4^ MRC Centre for Regenerative Medicine, University of Edinburgh, Edinburgh, United Kingdom.; ^5^ Advanced Materials for Bioengineering Research (AMBER) Centre, Trinity College Dublin and Royal College of Surgeons in Ireland, Dublin, Ireland.

**Keywords:** resident macrophages, cardiac macrophages, myocardial infarction, iPSCs, engineered heart tissue

## Abstract

Many facets of tissue engineered models aim at understanding cellular mechanisms to recapitulate *in vivo* behavior, to study and mimic diseases for drug interventions, and to provide a better understanding toward improving regenerative medicine. Recent and rapid advances in stem cell biology, material science and engineering, have made the generation of complex engineered tissues much more attainable. One such tissue, human myocardium, is extremely intricate, with a number of different cell types. Recent studies have unraveled cardiac resident macrophages as a critical mediator for normal cardiac function. Macrophages within the heart exert phagocytosis and efferocytosis, facilitate electrical conduction, promote regeneration, and remove cardiac exophers to maintain homeostasis. These findings underpin the rationale of introducing macrophages to engineered heart tissue (EHT), to more aptly capitulate *in vivo* physiology. Despite the lack of studies using cardiac macrophages *in vitro*, there is enough evidence to accept that they will be key to making EHTs more physiologically relevant. In this review, we explore the rationale and feasibility of using macrophages as an additional cell source in engineered cardiac tissues.

**Impact statement:**

Macrophages play a critical role in cardiac homeostasis and in disease. Over the past decade, we have come to understand the many vital roles played by cardiac resident macrophages in the heart, including immunosurveillance, regeneration, electrical conduction, and elimination of exophers. There is a need to improve our understanding of the resident macrophage population in the heart *in vitro*, to better recapitulate the myocardium through tissue engineered models. However, obtaining them *in vitro* remains a challenge. Here, we discuss the importance of cardiac resident macrophages and potential ways to obtain cardiac resident macrophages *in vitro*. Finally, we critically discuss their potential in realizing impactful *in vitro* models of cardiac tissue and their impact in the field.

## Introduction

Macrophages are present in almost every vertebrate tissue. They contribute to the maintenance of immunity by phagocyting microbes, clearing senescent cells, and promoting homeostasis by repairing and regulating the functions of organs. Initially perceived as a cell population only present in, and originating from, the blood, recent studies have brought to light the stark heterogeneity in blood-derived and pre-natal embryologically derived macrophage populations. Most tissues in the body contain a resident macrophage population exhibiting diverse phenotypes, specific to that tissue and niche.^1^ This phenotypic heterogeneity is potentially due to the impact of the resident tissue environment as well as to differences in the macrophage origin.^2^ However, despite being best known as the key mediators in tissue defense, the exact roles of different subsets of resident macrophages in protecting the body from pathogens or environmental change are still unclear, as they are hard to extract, purify, and study *in vitro*.

The focus of this review, cardiac resident macrophages, originates during embryogenesis and populates the heart prenatally.^3^ Only within the past 3–4 years, seminal studies unravelling the functions and the physiological importance of cardiac macrophages have emerged (in mice), identifying these cells as a potential candidate in tissue engineering applications. In addition to performing critical functions in healthy myocardium, cardiac resident macrophages respond uniquely to subtle changes in the homeostatic equilibrium. Although there have been a limited number of studies focusing on the role of resident macrophage populations in the heart, there exists enough groundwork to explore the possibilities of achieving more humanized cardiac tissue models using resident macrophages. In addition, by incorporating resident macrophages within engineered heart tissues (EHTs), models can be more physiologically relevant, with applications in regenerative medicine, disease modeling, and drug screening. However, there remain a lot of questions to be answered: What degree of these cells is lost during myocardial disease/ischemia? How do they function in response to injury and disease? What role do their paracrine factors exert in tissue engineered models, to name but a few?

We discuss cardiac macrophages in healthy myocardium, and during myocardial infarction (MI) and post-MI heart failure. We do a recap on the various EHT models that have emerged over the years and discuss the potential applications of macrophages within such models in cardiac tissue engineering. Finally, we discuss the challenges of obtaining cardiac resident macrophages *in vitro*, and avenues of obtaining them *in vitro* for tissue engineering, such as using induced pluripotent stem cell (iPSC) derived macrophages (iMacs), opening up the possibility to study genes and genetic pathways involved in their function.

## Macrophage Populations in the Heart and Their Functions

**FIG. 1.**
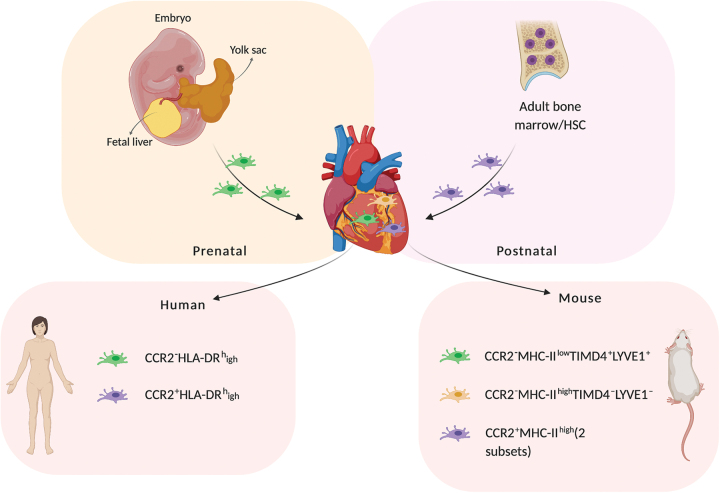
Macrophage subsets in human and mouse and their origin. Cardiac macrophages populate the heart prenatally from the embryonic yolk sac, whereas bone marrow-derived macrophages enter the heart 2 weeks after birth. In humans, the macrophage populations are distinguished based on the presence of CCR2, as CCR2^−^HLA-DR^high^ (cardiac macrophages) and CCR2^+^HLA-DR^high^ (bone marrow-derived macrophages). The three distinct populations of macrophages in the murine heart are CCR2^−^MHC-II^low^ (cardiac macrophages), CCR2^−^MHC-II^high^ (derived postnatally from cardiac macrophages), and CCR2^+^MHC-II^high^ (bone marrow-derived macrophages). *Created with BioRender.com* Color images are available online.

The cellular content of human myocardium (in descending order by number) predominantly consists of cardiomyocytes, fibroblasts, pericytes and smooth muscle cells, endothelial cells, and immune cells.^4^ In the murine heart, macrophage populations can be discerned based on their expression of C-C chemokine receptor type 2 (CCR2), major histocompatibility complex-II (MHC-II), T cell immunoglobulin and mucin domain containing 4 (TIMD4), and Lymphatic Vessel Endothelial Hyaluronan Receptor 1 (LYVE1). Four populations of macrophages have been identified in the murine heart^5^ (Fig. 1). The minority are two subsets of CCR2^+^MHC-II^high^ macrophage populations derived from hematopoietic stem cells, whereas the most dominant subset, cardiac resident macrophages derived from the yolk sac, are CCR2^−^MHC-II^low^TIMD4^+^LYVE1^−^. There also exists a fourth subset that consists of CCR2^−^MHC-II^high^TIMD4^+^LYVE1^+^ macrophages, which are likely derived from CCR2-MHC-II^low^ macrophages during postnatal development^5,6^ and partially maintained by CCR2^+^MHC-II^high^ macrophages. CCR2^+^MHC-II^high^ macrophages populate the heart soon after birth and are maintained through monocyte recruitment and proliferation.^7^ Also, under homeostasis, cardiac macrophages continually self-renew with a turnover period of almost 5 weeks.^8^ Recently, this same CCR2^+^ and CCR2^−^ macrophage distinction has been identified in humans,^9^ with the yolk sac-derived CCR2^−^ human counterpart showing CD14^+^CD45^+^CD64^+^CCR2^−^HLA-DR^high^ expression.^5,9^ In a more recent study, Tucker *et al.* reported two cardiac macrophage subsets within the human heart, which were positive for *CD163, COLEC12*, mannose receptor *MRC1*, E3 ubiquitin ligase *MARCH1*, and natural resistance–associated macrophage protein 1 (*NRAMP1*). Both of the identified subsets expressed anti-inflammatory (M2) polarization–associated genes, whereas one subset particularly expressed *RBPJ, F13A1* and the other expressed transmembrane collagen *COL23A1*.^10^

CCR2^+^ and CCR2^−^ macrophage populations are present in specific locations within the heart, performing distinct functions. CCR2^+^ macrophages are usually found in the trabecular projections of the heart, and their role in cardiac development has not been fully delineated. However, they are activated when homeostatic balance is interrupted, such as during a disease.^11^ In contrast, CCR2^−^ macrophages, herein referred to as cardiac resident macrophages, occupy the myocardial wall and play a principal role in normal coronary development and maturation.^12^ However, such studies are mostly limited to murine models. In addition to demonstrating robust phagocytic behavior,^13^ cardiac resident macrophages have demonstrated a role in neonatal cardiac regeneration in mice, salamanders, and zebrafish,^14–16^ potentially through the stimulation of angiogenesis.^17^

**FIG. 2.**
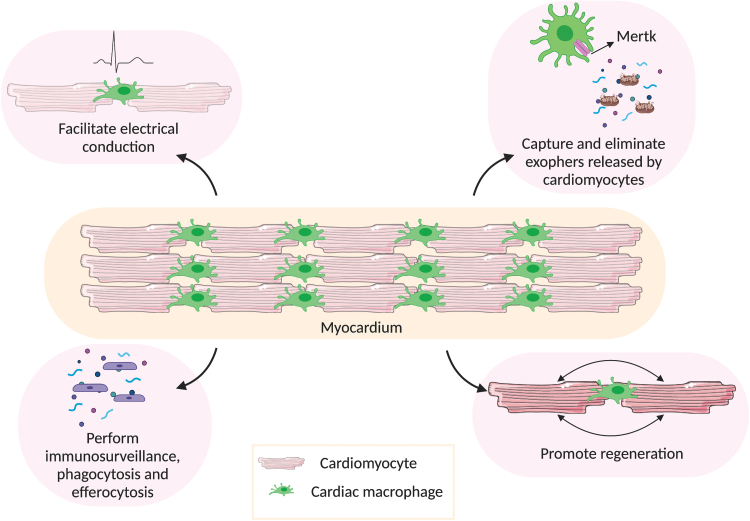
Functions of cardiac macrophages in healthy myocardium. In steady state, cardiac macrophages perform several important functions in the myocardium. One of their major functions is to facilitate electrical conduction by coupling with cardiomyocytes through connexin-43 gap junctions. They help in sustaining normal cardiac autophagy by capturing and eliminating exophers (containing defective mitochondria) released by cardiomyocytes, through Mertk. They also perform immunosurveillance and clear dead cells and debris in the myocardium, to maintain homeostasis. The presence of cardiac macrophages is found to be essential in promoting cardiac regeneration. *Created with BioRender.com* Color images are available online.

Further, cardiac resident macrophages interact very closely with cardiomyocytes and form signaling complexes through connexin-43, allowing synchronous depolarization^18^ (Fig. 2). Hulsmans *et al.* have shown that depletion of either cardiac resident macrophages or connexin-43 effects a delay in conduction and blockage in the atrioventricular node in mice. This study highlights a critical role of cardiac macrophages in coupling with cardiomyocytes to facilitate electrical conduction within the heart. Undoubtedly, the coupling of cardiomyocytes and cardiac resident macrophages holds a lot of possibilities in regenerative medicine, to be explored in the future.

In addition to their involvement in the conduction system of the heart, cardiac macrophages also play a role in phagocytosis and efferocytosis^19^ (Fig. 2). Cardiac macrophages express high levels of myeloid epithelial reproductive tyrosine kinase (Mertk), which plays a role in macrophage-mediated efferocytosis. In the absence of this protein, cell debris removal during cardiac repair is severely affected, resulting in increased infarct size and reduced cardiac function.^20^ The removal of debris and other materials ejected by cardiomyocytes is crucial in maintaining adequate cardiac function, even in non-pathological settings. Similar to how the central nervous system disposes of dysfunctional organelles,^21^ cardiomyocytes remove dysfunctional subcellular particles with the help of cardiac resident macrophages.^22^ Likewise, Nicolás-Ávila *et al.* documented that cardiomyocytes, which are continuously contracting and metabolically active cells, release subcellular particles termed “exophers” containing defective mitochondria.^22^ These exophers are taken up by cardiac resident macrophages through Mertk to maintain cardiac homeostasis. This maintenance role of cardiac resident macrophages is further demonstrated when they are depleted in the myocardium (by an intraperitoneal injection of 10 μg/kg diphtheria toxin in mice),^22^ which, in turn, reduces the presence of these exophers, delineating the role of cardiac resident macrophages in maintaining normal cardiac autophagy. In addition to unveiling this unique role of cardiac resident macrophages, this is the first study to show that myocardial tissue releases exophers (in mice).

## Macrophage Populations During MI and Post-MI Heart Failure

**FIG. 3.**
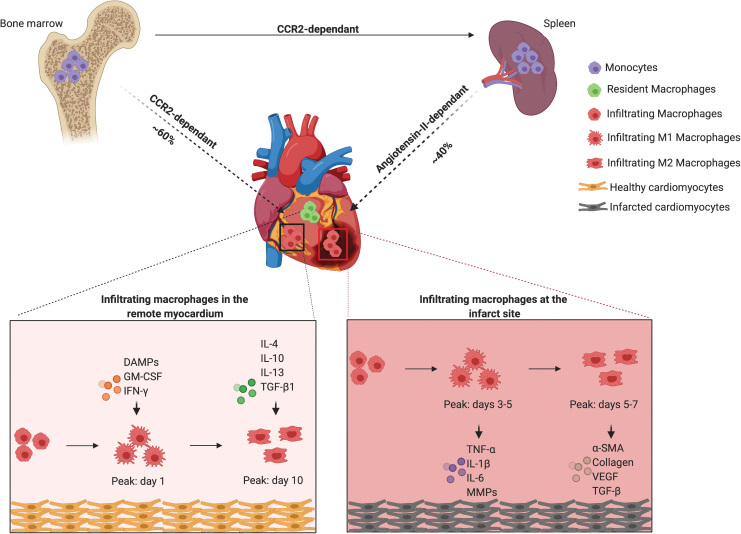
Macrophage origin and function in infarcted heart. After MI, the monocytes that infiltrate the heart originate from bone marrow (∼60%) and spleen (∼40%). The recruitment of bone marrow-derived monocytes is CCR2 dependent and that of spleen-derived monocytes is angiotensin II-dependent. Cardiac resident macrophages reside, self-renew, and carry out a number of functions in the myocardium. However, at the infarct site and remote myocardium, infiltrating macrophages differentiate to M1 and M2 macrophages in response to different cues, and they secrete pro-inflammatory and pro-healing factors based on their macrophage phenotype. The number of M1 and M2 macrophages peak in the infarct site and remote myocardium at a different pace. *Created with BioRender.com* Color images are available online.

In the murine heart, the macrophages and monocytes population in the myocardium after an infarct is dramatically different from that in steady state (Fig. 3). The temporal dynamics of leukocyte infiltration after surgically induced MI has been assessed by using flow cytometry and determines that neutrophils are among the first leukocytes to reach ischemic heart tissue.^23^ This migration of leukocytes to the ischemic site is mediated by C-X-C motif chemokine ligand 2 (CXCL2) and CXCL5 production by CCR2^+^ macrophages.^24^ Monocyte recruitment to the infarct site begins as early as 30 min depending on CCR2 signaling, from both the bone marrow and the spleen, via MCP-1/CCR2 signaling.^25^ This infiltration of monocytes and macrophages to the infarcted tissue occurs in two phases.^23^ The Ly-6C^high^ monocytes exhibiting pro-inflammatory phenotype predominate the early days (marking the first phase), peaking on day 3^23^ (Fig. 3). Around 40% of the infiltrating Ly-6C^high^ monocytes in the first 24 h post-MI are from the splenic monocyte reservoir.^26^ During this period, also known as the inflammatory phase, macrophages and neutrophils clear dead cells and debris. After proper resolution of the inflammatory phase, there occurs a phenotypic transition from Ly-6C^high^ monocytes to Ly-6C^low^ monocytes and M2 macrophages, which exert reparative functions and renew locally.^26^ Ly-6C^low^ macrophages predominate the later stages as inflammation resolves (marking the second phase), peaking between days 5 and 7 post-infarct^27^ (Fig. 3). Similarly, the remote myocardium also undergoes a sequential Ly-6C^high^ to Ly-6C^low^ differentiation pattern, with the Ly-6C^low^ cells peaking 5 days later than at the infarct.^28^ The cardiac resident macrophages in the infarct area die shortly after MI.^6^ However, more recent studies show that in the post-MI phase, the majority of the cardiac resident macrophages proliferate locally with a minor contribution from blood-derived monocytes.^6^

During the inflammatory phase, classically activated (M1) macrophages secrete cytokines such as tumor necrosis factor α (TNFα), interleukin (IL)-1β, IL-6, and matrix metalloproteinases (MMPs) to facilitate extracellular matrix (ECM) breakdown.^29^ Phagocytosis mediated by M1 macrophages is crucial for the initiation of the wound healing process. However, prolonged production of these inflammatory cytokines could lead to a number of pathological consequences, including cardiomyocyte death, cardiac rupture, fibrosis, dilated cardiomyopathy due to matrix degradation etc.^30^ For example, TNFα (produced by macrophages, cardiomyocytes, and endothelial cells) induces cardiomyocyte hypertrophy, which can lead to post-MI heart failure.^31^ Over the next few days, macrophages promote fibroblast differentiation into myofibroblasts, which express smooth muscle actin and lay down collagen, contributing to scar formation.^32^ In addition, vascular endothelial growth factor (VEGF) and transforming growth factor-β (TGF-β) produced by the macrophages facilitate revascularization of tissues.^23,33^ Finally, macrophages also produce MMPs that help in breaking down the ECM and promote tissue remodeling.

Any abnormalities in either of the phases can be detrimental to the cardiovascular system. In particular, macrophage depletion is deleterious to infarct healing and worsens the remodeling process.^34,35^ Despite being merely 2–5% of the total macrophage population in the heart in the initial weeks post-MI, the depletion of cardiac resident macrophages impaired normal infarct healing and adversely affected the remodeling process.^5^ Shiraishi *et al.* demonstrated the effect of inactivation of alternatively activated (M2) macrophages post-MI by using a mouse model with Trib1 gene deletion.^36^ The impaired wound remodeling was rescued by the introduction of M2 macrophages exogenously. In addition, limiting the number of infiltrating monocytes via CCR2 blockage has shown to decrease the infarct size in MI recovery animal models,^37–39^ demonstrating that the relevance of therapeutic approaches to limit infiltrating monocyte numbers is patients with increased systemic inflammatory activity to improve post-MI recovery.

As previously mentioned, initial fibrosis after MI is necessary to facilitate scar formation and initiate tissue remodeling. However, extensive fibrosis can result in morbid cardiac remodeling and heart failure.^40^ The macrophage number in the heart progressively increases from the onset of MI to heart failure, with local proliferation as well as recruitment responsible.^41^ Although macrophages in the remote myocardium increase, their number depletes at the site of infarct.^6^ This local expansion of cardiac macrophages aggravates the progression of heart failure by producing factors that promote fibrosis, such as TGF-β, platelet-derived growth factor, and angiotensin-II.^42^ Myofibroblasts and cardiomyocytes also further prompt TGF-β production, causing adverse remodeling that leads to heart failure. The detrimental effects of recruited monocytes/macrophages are further recognized, as splenectomy reduces infiltrating macrophages and improves cardiac function.^43^ A recent report also suggested that mechanical strain can trigger macrophage proliferation by activating the mitogen-activated protein kinase (MAPK) pathway, demonstrating that increased higher mechanical strain in the failing heart could stimulate macrophage expansion.^6^ This evidence further highlights the ill impacts of infiltrating macrophages in the infarcted heart. It is also of importance to note recent studies, suggesting that the opportunity for successful regeneration of the heart after MI is restricted to 3 days post-MI and is potentially mediated by cardiac resident macrophages and fibroblasts.^44^

## EHT Models

Tissue engineering considerably enables our understanding of disease biology and possibilities for therapeutic intervention.^45^ As opposed to 2D cell culture models, tissue engineered cardiac models provide the benefit of mimicking *in vivo* myocardium, with the additional functional benefit of tissue contraction, which is inherent to cardiac function.^46^ Moreover, it also comes with the ethical benefit of using a lesser number of animals. Engineered constructs are typically generated by using primary cardiomyocytes (adult, neonatal, fetal), as well as cardiomyocytes obtained through iPSCs or embryonic stem cells (ESCs).^47^ Although EHTs can be fabricated purely by using cardiomyocytes, the contractile function and the complexity of native myocardium is better recapitulated with the addition of non-myocytes, such as fibroblasts and endothelial cells.^48^ Other non-myocytes that have been explored include cardiac stem cells.^49^

**FIG. 4.**
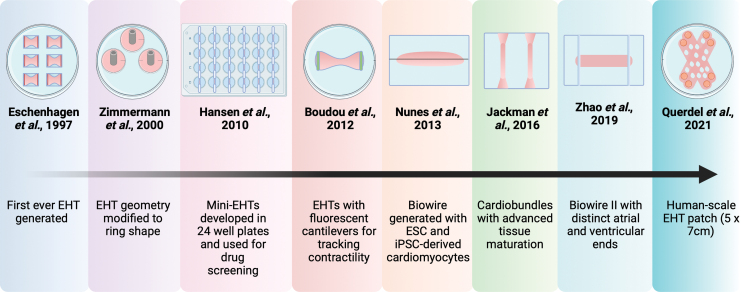
Brief overview of the evolution of Engineered Heart Tissues. The very first EHT was generated in 1997 by using chick embryonic cardiomyocytes on Velcro-coated glass tubes.^50^ This model and the geometry of EHTs evolved over the years, from ring-shaped EHTs^51^ to mini-EHTs adapted to a 24-well plate format,^53^ to EHTs with fluorescent cantilevers that can track contractile motions,^54^ followed by Biowire,^56^ cardiobundles,^58^ and Biowire II,^59^ to finally reach a human-scale EHT patch.^60^ EHT, engineered heart tissue. *Created with BioRender.com* Color images are available online.

More than two decades ago, the first EHT model was fabricated with chick embryonic cardiomyocytes mixed with Matrigel, which anchored on to opposite ends of two Velcro-coated glass tubes^50^ (Fig. 4). This culture provided mechanical strains to the engineered tissue and, hence, was utilized as a platform for understanding the effects of uniaxial and multiaxial stretching, growth factors, cardiomyocyte maturation, etc. This same principle was used to modify the geometry of the EHT to obtain a ring-shaped model that supported mammalian (rat) cardiomyocytes and also facilitated long-term force measurements.^51,52^ Although a number of different scaffold-based cardiac tissue engineered models were developed around this period, the next major modification to the EHT design was miniaturized EHTs made with fibrin, which were adapted to a 24-well format, to facilitate drug screening.^53^ This study demonstrated the use of video-optical recording for the measurement of contractility of the tissues, which is a commonly used method of contractility measurement at the moment.^53^ Shortly after, Boudou *et al.* generated collagen/fibrin matrix-based cardiac microtissues by using microfabricated tissue gauges that incorporate microelectromechanical systems cantilevers.^54^ This facilitated in simultaneously constraining and reporting forces generated by the tissues in real time.^54^ Similar approaches with cantilevers to allow tissue anchorage have also been used by Turnbull *et al.* to facilitate fabrication of potential preclinical *in vitro* myocardium models.^55^ Another interesting modification to the EHT came with the use of a surgical suture within PDMS molds to obtain aligned cardiac tissues with striations called “Biowire.”^56^ After this study, Bian *et al.* demonstrated that fabricating cardiac tissues with longer elliptical pores within the patch networks improve ECM alignment within the patch.^57^ In the past 5 years, the field of EHTs further underwent drastic advances where fibrin-based EHTs under dynamic culture (using platform rockers) showed improvement in tissue maturation.^58^ These EHTs, called “cardiobundles,” were designed to be cylindrically shaped and anchored within porous flexible nylon frames that supported chronic auxotonic loading and free-floating culture of the tissues.^58^ More recently, the advanced successor of Biowire, named “Biowire II,” a heteropolar cardiac tissue containing distinct atrial and ventricular ends was generated.^59^ This heteropolar tissue formation was achieved by using directed cell differentiation and electrical field conditioning.^59^ With more than two decades of input, one of the most recent advancements in EHTs is a mesh-structured EHT patch of dimension 5 × 7 cm, which closely resembles human ventricular tissue.^60^

One of the key features of EHTs is their ability of responding to drugs. Most of the different types of EHTs fabricated over the years aim at featuring their drug response. For example, more than a decade ago, Hansen *et al.* showed that EHTs display a concentration-dependent increase in relaxation time on treatment with chromanol, quinidine, and erythromycin, whereas doxorubicin treatment resulted in decreased contractile force.^53^ Similarly, Boudou *et al.* tested isoproterenol and digoxin on their EHTs to understand the functional consequences of pharmacologic agents.^54^ They showed that small doses (10 and 100 nM) of isoproterenol increased the contractility of the EHTs whereas 1 and 10 mM decreased the contractility.^54^ On the other hand, digoxin improved the contractility at 1 and 1 mM but it was found to be cardiotoxic at 10 mM concentration.^54^ Hence, the varied and dose-dependent sensitivity of EHTs toward different drugs makes them an exciting alternative to animal models, in addition to being a useful tool in understanding the effect of various drugs on the myocardium. Despite the proven efficacies of these EHTs, there still remain challenges in making them more humanized, including the addition of right cell types to mimic the complexity of the myocardium. For a more detailed review capturing EHT, the reader is referred to the paper by Christian Zuppinger.^61^ One of the potential additions to improve the response of EHTs would be cardiac resident macrophages, as they are critical in the normal functioning of the heart.

## Macrophages in Tissue Engineering

In recent years, the critical importance of incorporating macrophages into engineered constructs is being recognized^62,63^ and aside from the immunological responses of host macrophages, their role has been harnessed in a number of tissue engineering approaches to improve tissue maturation, repair, and regeneration.^64^ Understanding macrophage responses during biomaterial design,^65,66^ as well as exploring the effects of mechanical cues on macrophage polarization are other areas within tissue engineering that have gained attention.^67,68^ Researchers have studied the potential of immune-assisted tissue engineering, by incorporating macrophages into tissue engineered constructs (Table 1). In particular, macrophages have been used to vascularize biomaterials,^69^ by promoting angiogenesis^70^ and vessel formation.^71^ Although immune-assisted tissue engineering has not been largely investigated, these data show the significance of macrophages in various tissue engineered constructs.

**Table 1.** Examples of Macrophage Use in Tissue Engineering

Tissue	Macrophage	Application
Bone	Blood-derived macrophages	Vascularization of scaffold^69^
	Blood-derived macrophages	Osteogenesis^102,103^
Skeletal muscle	Blood-derived macrophages	Regeneration^63^
Blood vessel	Blood-derived macrophages	Vascularization of scaffold^71^
Liver	Kupffer cells	Hepatotoxicity^104^
	Kupffer cells	Disease-drug interaction^105^
Nerve	Microglia	Regeneration^106^
Brain/spinal cord	Microglia	Understand cellular crosstalk during neuroinflammation^107^
Lungs	Alveolar macrophages	Understanding response to atmospheric particles^108^
	Alveolar macrophages	*In vitro* lung inflammation platform^109^
	Alveolar macrophages	*In vitro* model for air–blood barrier^110^

Within cardiac tissue engineering, the specific functional requirement of this tissue includes contraction, which is inherent to cardiac function.^46^ As macrophages have been shown to be integral during signal propagation (Fig. 2), their inclusion in engineered tissue could facilitate robust and more physiologically accurate contraction mechanics. Engineered constructs are typically generated by using primary cardiomyocytes (adult, neonatal, fetal), as well as cardiomyocytes obtained through iPSCs or ESCs.^47^ Since the critical role played by macrophages in cardiac regeneration, the maintenance of homeostasis and disease progression has been recognized (in mice), cardiac tissue engineering has begun to focus on incorporating macrophages into EHTs to recapitulate native tissue functions. A pioneering *in vitro* study in which human peripheral blood-derived macrophages and iPSC-derived cardiomyocyte interaction was assessed attempted to recapitulate critical cellular events during myocardial injury, using a 3D inversion assay. They proposed a bone morphogenetic protein (BMP) mediated crosstalk between these two cell types.^72^ Cardiomyocyte-derived BMP promoted pro-inflammatory (M1) macrophage recruitment, and M1 macrophage-derived BMP improved cardiomyocyte proliferation and differentiation potential. This proposed paracrine mechanism of BMPs was new and provided a new insight into the mechanism of interaction between cardiomyocytes and peripheral blood-derived macrophages. Interestingly, a recent study has shown that the presence of macrophages in engineered cardiac tissue facilitates cardiomyocyte de-differentiation and remodeling.^73^ In this study, it was observed that during the initial days (up to 3 days), the macrophages in the engineered cardiac tissue were polarized to an M1 state (CD86 positive), followed by a predominance of M2 macrophages (CD206 positive) as the cardiomyocytes de-differentiated. The effects of macrophage activity on the engineered cardiac tissue model could not be explained in this study, however, as a heterogenous population of whole cells from rat heart was used to make the engineered cardiac tissue. Nonetheless, future studies into the influence of macrophage populations of different ontological origin on cardiac function will shed light on their potential role in cardiac tissue engineering.

One study by Wrona *et al.* explored the response of human hESC-derived cardiomyocytes to peripheral blood-derived monocytes, with a focus on investigating the effects of polarized macrophages and their activation inducing cytokine profiles on cardiomyocyte function.^74^ In this study, the gene expression profile of the macrophages remained unaffected on exposure to hESC-derived cardiomyocytes whereas cardiac-specific genes were downregulated on exposure to M1 and M2 polarized macrophages.^74^ Tying in with the findings of this work, a study by Hitscherich *et al.* using murine ESC-derived cardiomyocytes also showed a decrease in the expression of cardiac-specific genes such as cardiac troponin T and sarcoplasmic/endoplasmic reticulum calcium ATPase when cultured with conditioned media from M1 polarized blood-derived murine macrophages.^75^ Here, a coculture system was employed, to demonstrate that M1 macrophages promoted a higher Ca^2+^ fractional release and both macrophage subtypes downregulated the store-operated Ca^2+^ entry in murine ESC-derived cardiomyocytes. As previously discussed, EHTs and other scaffold-based tissue engineered models for the myocardium heavily rely on cardiomyocytes, cardiac fibroblasts, and endothelial cells to represent the myocardium. Although literature shows the potential of EHTs in guiding us toward obtaining robust *in vitro* models of human myocardium that has the ability to mature, beat in synchrony, and respond to drugs, the addition of macrophages into these models could benefit our understanding on the *in vitro* behavior of macrophages in a complex, multicellular humanized setting. From the available *in vivo* studies (in mice), it could be anticipated that the incorporation of cardiac resident macrophages within an EHT (containing cardiomyocytes) will facilitate improved contractility and electrical conduction of the tissue. In addition, the tissue could be hypothesized to exhibit improved maturity as there could be constant exophers elimination and maintenance of homeostasis.

Another interesting avenue that can be pursued by using an EHT model incorporates cardiac resident macrophages is screening anti-inflammatory drugs for post-MI treatment. Although clinical studies on drug treatment in patients with MI are limited, there are a number of drugs that have been developed for regulating immune response post-MI.^76^ This includes non-steroidal anti-inflammatory drugs, glucocorticoids, cyclosporine etc.^77^ Despite showing an improvement in mortality, these broad anti-inflammatory drugs often fail to provide a cardioprotective effect.^77^ To circumvent this limitation, targeted anti-inflammatory drugs can be used. For example, the CANTOS (Canakinumab Anti-Inflammatory Thrombosis Outcomes Study) trial was the first large cardiovascular clinical trial to target the inhibition of inflammation by using the drug canakinumab, which inhibits IL-1β to subsequently reduce the production of IL-6 and high sensitivity C-reactive protein (hsCRP).^78^ Among the 10,061 patients with previous MI and hsCRP level ≥2 mg/L, patients treated with 150 and 300 mg of canakinumab experienced a 15% reduction in major adverse cardiovascular events in comparison with placebo.^78^ Although canakinumab was not ultimately approved for cardiovascular treatment, CANTOS trial demonstrated that targeting the inflammatory pathway involving IL-1β and hsCRP reduces adverse cardiovascular events. CANTOS was followed by CIRT (Cardiovascular Inflammation Reduction Trial), in which 4786 patients with stable atherosclerosis with diabetes mellitus or metabolic syndrome were subjected to low-dose methotrexate.^79^ Low-dose methotrexate failed to reduce major adverse cardiovascular events, and it also failed to reduced plasma levels of IL-1β and hsCRP, further demonstrating the importance of adequate inhibition of the IL-1β to IL-6 pathway for long-term cardiovascular benefits.^80^ Further large cardiovascular outcome trials targeting the inflammatory pathways that followed include COLCOT (Colchicine Cardiovascular Outcomes Trial)^81^ and LoDoCO2 (Low Dose Colchicine after MI).^82^ Both of these trials used low doses of colchicine (0.5 mg/day), which is a repurposed anti-inflammatory agent. Although COLCOT had 4745 patients with recent MI (less than 30 days), LoDOCO2 had 5522 patients with chronic coronary artery disease and both the studies showed a reduction in the incidence of adverse cardiac events such as MI, stroke, cardiovascular death etc.^81,82^ However, colchicine, being associated with an increase in pneumonia, nausea, and flatulence, poses risk.^80^ To that end, the lack of success for most of these drugs could also stem from the fact that they are not thoroughly validated *in vitro* before human trials. The trajectory of recent and ongoing cardiovascular outcome trials shows an increased interest toward novel anti-inflammatory and anti-cytokine drugs.^80^ This further demonstrates the benefit of an *in vitro* model of the myocardium that has innate immune cells (especially macrophages) as they serve as the primary target for these drugs and can potentially fill the gap between the patient and the pharmaceutical industry.

## iPSC and ESC-Derived Macrophages

**FIG. 5.**
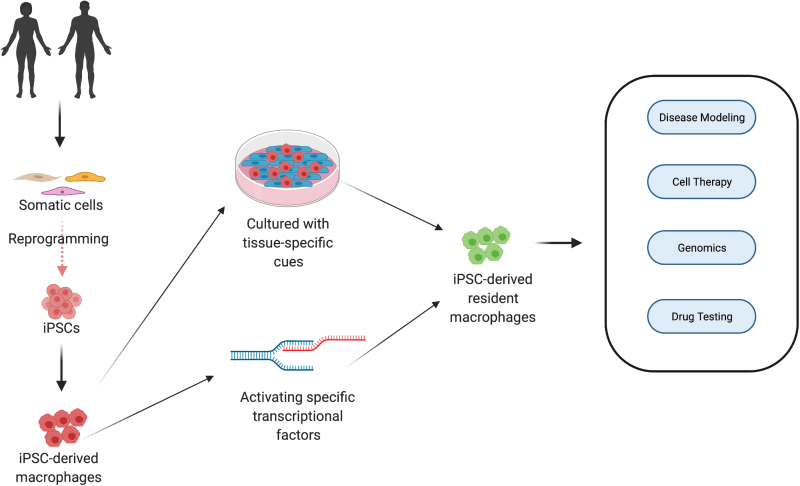
Macrophage derivation from iPSCs and their applications. The iPSCs can be derived from human beings by reprogramming somatic cells (fibroblasts, blood cells etc.). On specifying the mesodermal origin, they can be differentiated into macrophages. Cardiac resident macrophages can potentially be obtained by giving iPSC-derived macrophages tissue-specific cues or activating specific transcriptional factors. These macrophages can be used for a number of applications, such as disease modeling, cell therapy, genomics (to identify new genes), and drug testing. iPSC, induced pluripotent stem cell. *Created with BioRender.com* Color images are available online.

Obtaining human cardiac macrophages for subsequent *in vitro* studies is challenging. To date, macrophage studies have heavily relied on murine or human blood-derived monocytes but these cells are not ideal for appropriating tissue resident phenotypes due to their vast transcriptomic and epigenetic variations, as well as difference in origin between blood-derived and resident macrophages.^83^ Obtaining primary resident macrophages from vital organs (cardiac macrophages, Kupffer cells, microglia etc.) is not feasible, as acquiring tissue samples from healthy donors is often associated with donor site morbidity, in addition to ethical considerations. To circumvent these limitations, the use of iPSCs and hESCs as macrophage progenitor sources has come to the forefront (Fig. 5 and Table 2). Over the past decade, a number of different protocols have been established for stem cell culture and differentiation, to obtain CD14^high^CD16^low^CD163^+^CD11b^+^ macrophages, morphologically similar to blood-derived macrophages.^84–88^ Critically, iPSC and hESC-derived macrophage sources have shown therapeutic benefits in ameliorating liver fibrosis,^89^ acute lung injury^90^ and they are also under clinical trial for liver cirrhosis.^91^

**Table 2.** Examples of Induced Pluripotent Stem Cell-Derived Macrophage Subsets and Their Respective Phenotypic Markers

Tissue	Cell-type	Phenotypic markers
Blood	Blood-derived macrophages	CD18^+^, CD11b^+^, CD11c^+^, CD14^+^, CD16^+^, CD115^+^, CD1a^−^, CD83^−^, CD3^−^, CD19^− 111^
		CD14^+^, CD16^+^, CD163^+^, CD86^+ 112^
		CD45^+^, CD11b^+^, CD14^+^, CD163^+^, CD68^+ 113^
Lungs	Alveolar macrophages	CD80^+^, SIRPa^+^, MHCII^−^, Langerin^− 90^
Liver	Kupffer cells	CLEC-4F^+^, ID1^+^, and ID3^+^
Brain	Microglia	IBA1^+^, CD11c^+^, TMEM119^+^, P2RY12^+^, CD11b^+^, and CX3CR1^+ 114^
		CD11b^+^, ITGB2^+^, CSF1R^+^, CD45^+^, IBA1^+^, and ADORA3^+ 115^

Further, recent studies have shown that iMacs share ontogeny with tissue resident macrophages and are independent of MYB,^90,92,93^ suggesting that iMacs in isogenic cocultures may offer a facile and effective approach to obtaining physiologically relevant resident macrophages.^94^ For example, iMacs in coculture with neural cells were found to become microglia-like cells.^95,96^ Haenseler *et al.* showed that iMacs in coculture with iPSC-derived neurons expressed markers relevant to microglia as well as major neurodegenerative disorders.^84^ Similarly, iMacs in coculture with hepatocytes were found to show Kupffer cell-like characteristics.^93^

In addition to coculture models, the effect of the local biochemical environment on the specialization of tissue-resident macrophages is becoming more evident in regenerative medicine. Certain transcriptional factors regulate the expression of tissue-resident macrophages,^97^ such as Spi-c for the development of the red pulp macrophages in the spleen,^98^ Sall1 for microglia,^99^ and Id3 for Kupffer cells.^100^ In a more recent study, the activation of iMacs with transcriptional factor KLF1 was found to drive them toward an erythroblastic island-like phenotype.^101^ These studies demonstrate that physiologically relevant resident macrophages could be obtained *in vitro* by providing tissue-specific cues or by activating-specific transcriptional factors. Although cardiac resident macrophages have not yet been derived from iPSCs, research is proceeding in the right direction, paving the way to understanding optimal methods of cardiac resident macrophage acquisition *in vitro*, such as using primary cardiomyocyte conditioned media on iPSC-derived macrophages, establishing coculture models of cardiomyocytes and iPSC-derived macrophages, or using bioinformatics tools to determine transcriptional factors for their induction. Taken together, iPSC-derived macrophages offer great potential in understanding and modeling tissue resident macrophages.

## Conclusion

From regarding macrophages as a system of clearing dead cells, to appreciating their role in tissue defense, homeostasis, regeneration, and organ development, our understanding of macrophages has advanced significantly in recent years. Lineage tracing and fate mapping studies demonstrated that most vital organs are largely populated by resident macrophages at the onset of embryological development and play critical roles in health and disease. Within the heart, resident macrophages ensure homeostatic cardiac function by performing a number of vital functions, including phagocytosis, efferocytosis, eliminating exophers, facilitation of electrical conduction etc. Our understanding of cardiac immunology has progressed well enough to regard cardiac resident macrophages as a potential candidate to include in EHTs, to further our understanding of cell interactions in addition to making the engineered tissues more physiologically relevant. However, much more still remains to be understood, such as what degree of these cells are lost during myocardial disease/ischemia; how do they function in response to injury and disease, their paracrine effects in tissue engineered models etc. The next step in cardiac tissue engineering is to unravel the crosstalk between cardiac macrophages and cardiomyocytes *in vitro* and further our understanding of the molecular processing involved in their interaction and their potential in regenerative medicine, therapeutics, disease modeling, and drug screening. With EHTs gaining great research interest, it is highly pertinent to include a resident macrophage population to make the tissue more physiologically relevant. By building on our strong foundational knowledge on different macrophage populations and with the assistance of new technologies, we will hopefully see rapid progress in finding new therapeutics using cardiac tissue engineering.
